# Spatial omics for profiling the dynamic tumor microenvironment

**DOI:** 10.1002/cti2.70084

**Published:** 2026-04-13

**Authors:** Hao Nguyen, Merrin Mary Eapen, Quan Nguyen, Ankur Sharma

**Affiliations:** ^1^ Queensland Institute of Medical Research, Berghofer Brisbane QLD Australia; ^2^ Translational Genomics Program Garvan Institute of Medical Research Darlinghurst NSW Australia; ^3^ School of Clinical Medicine, Faculty of Medicine and Health University of New South Wales Kensington NSW Australia; ^4^ Institute for Molecular Bioscience The University of Queensland Brisban QLD Australia

**Keywords:** artifical intelligence, cellular interactions, precision cancer medicine, spatial proteomics, spatial transcriptomics, tumor microenvironment

## Abstract

Spatial transcriptomics (ST) and spatial proteomics (SP) have revolutionised our ability to map RNA and protein distributions within intact tissues, shedding new light on the dynamic interactions that drive physiological processes in healthy and diseased tissues. We discuss how the latest ST and SP technologies, large public data resources and advanced computational pipelines can be applied to study the tumor microenvironment (TME), focussing on the interactions within the TME. We also highlight how these developments have enabled the in‐depth spatial characterisation of tumors and their TME across the continuum of cancer progression, from initiation to metastasis. Despite these advances, major gaps persist in cross‐platform integration, data standardisation and computational scalability for high‐plex single‐cell datasets. The integration of artificial intelligence (AI) holds great promise for biological and translational applications but requires standardised workflows, cost‐effective pipelines, rigorous pre‐clinical and clinical validation, and improved interpretability of AI models. Additional cross‐disciplinary development of explainable, scalable tools for TME analysis of cellular interactions and disease progression will be essential to integrate spatial omics into daily precision cancer medicine.

## Graphical abstract

Spatially resolved transcriptomics and proteomics map RNA/protein in intact tumor tissues, revealing crucial cancer–immune interactions across the different stages of tumor evolution. Integrating AI promises translational impact, but gaps in cross‐platform integration, standards, compute, validation and interpretability remain. Converging technologies support scalable, cost‐effective workflows towards routine precision oncology.

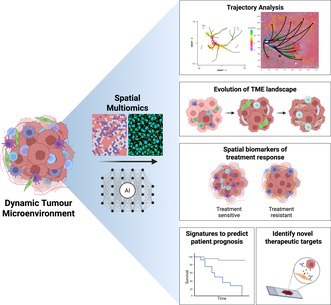

## Introduction

Cancers are highly complex and dynamic ecosystems in which malignant cells coexist with a diverse array of non‐malignant components within an altered extracellular matrix.^1^ The TME is highly heterogeneous across patients and tumor types, and its spatially organised niches and cell‐to‐cell communication can strongly influence tumor progression, immune infiltration and treatment response.^2^

Spatial omics technologies and computational tools have advanced significantly over the past decade. Spatial transcriptomic and proteomic technologies have enabled our ability to investigate the spatial dynamics of gene and protein landscape in the tissue context. They map the cellular state, gene expression patterns and ligand–receptor interactions within the intact tissue architecture. This unravels the disequilibrium in cell‐to‐cell communication in disease conditions such as cancer and captures the complexity of the TME in exceptional resolution.

As next‐generation sequencing (NGS) became more accessible, bulk RNA sequencing (bulk RNA‐seq) and single‐cell RNA sequencing (scRNA‐seq) were widely adopted into cancer research. Bulk RNA‐seq provides robust aggregate expression profiles across tissue samples, whereas scRNA‐seq allows for cellular‐level transcriptomic characterisation; the mechanistic dissociation of tissues leads to the loss of spatial information. However, both approaches have limitations in interrogating spatial context: bulk RNA‐seq averages signal across mixed cell populations, and scRNA‐seq typically requires tissue dissociation, which disrupts tissue architecture, removing information about cellular neighbourhoods and microenvironmental niches. However, studying the transcriptome and proteome within each cell's complex spatial niches offers insights into its interaction within the TME and its effects on tumor behaviour and clinical outcomes.

Recognising this need, spatial omics technologies are being widely adopted in cancer research. This summary comprehensively discusses how ST and SP technologies have produced valuable data and analytical tools and enabled the discovery of complex immunological processes within cancer TME. We also highlight the translational impact of spatial omics technologies, focussing on their applications in understanding the dynamics of cancer progression and emerging clinical implications.

## Advances in spatial transcriptomic and spatial proteomic technologies over the last decade

### Applying spatial transcriptomics to decipher the complex TME landscape

**Figure 1:**
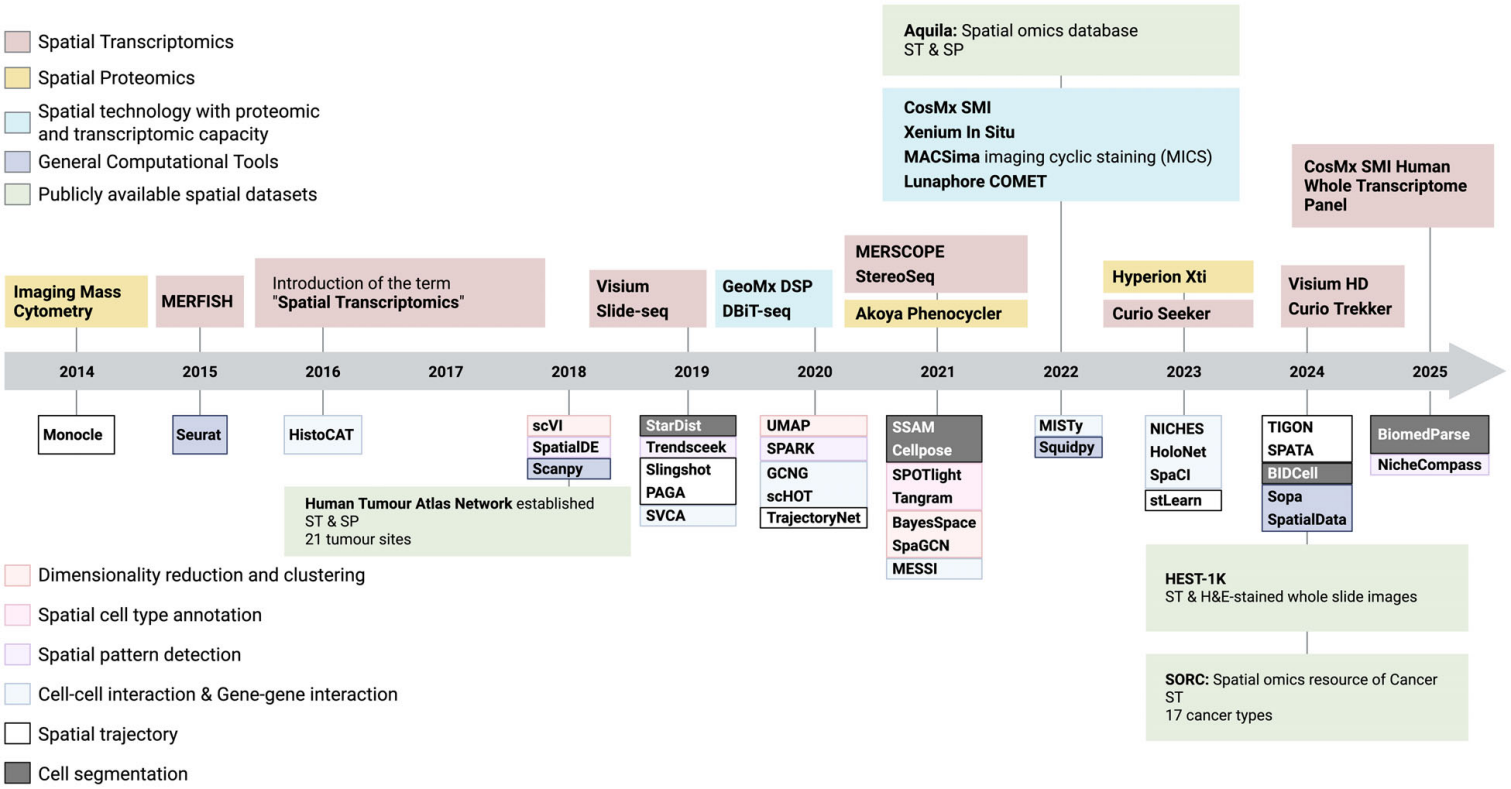
A timeline summarising advancements in spatial technologies and analytical tools. This timeline shows the main spatial transcriptomic and proteomic technologies developed over the past decade, along with large publicly available pan‐cancer spatial datasets. Since the introduction of the term “spatial transcriptomics “in 2016, experimental methodologies have evolved from low to high resolution methods and targeting a few proteins or genes to highly multiplexed and whole transcriptome approaches. Alongside experimental advancements, numerous analytical tools have emerged, expanding the scope of analysis of spatial data. The past decade has also witnessed the development of publicly available spatial datasets such as the establishment of the Human Tumor Atlas Network (HTAN) derived from multiple tumor types, providing a rich resource to drive cancer research. ST, spatial transcriptomics; SP, spatial proteomics; H&E, haematoxylin and eosin. This figure was created using Biorender.

The term ‘Spatial Transcriptomics’ was introduced in 2016 to describe an *in situ* hybridisation (ISH)‐based method that used spatially barcoded primers to introduce spatial coordinates to RNA‐sequencing data.^3^ These spatial barcodes were linked to reverse transcription oligo‐dT primers attached to glass slides, where the tissue sections were placed.^3^ Following permeabilisation, mRNA is reverse transcribed and sequenced.^3^ The sequenced transcriptomic data are analysed alongside the histological image of the tissue, with the spatial barcodes aiding in aligning each transcript back to its spatial location.^3^ ST has undergone significant improvements over the years, which have been complemented by the development of various computational tools to facilitate the analysis of the increasingly large and complex datasets (Figure 1).

### Sequencing and imaging‐based spatial transcriptomic technologies

Spatial transcriptomics includes sequencing‐based and imaging‐based approaches. Visium and Visium HD (10× Genomics) are sequencing‐based whole‐transcriptomic approaches, with Visium achieving 55 μM resolution covering 8–20 cells, and Visium HD with 2 μm single‐cell resolution.^4^, ^5^ Spatially enhanced resolution omics sequencing (Stereo‐seq), commercialised as STOmics (BGI Group), uses chips with DNA nanoballs (DNBs) containing unique coordinate identity (CID), barcodes (UMI) and poly‐T oligonucleotides.^6^ Slide‐seq, another sequencing‐based ST method (commercialised as Curio Seeker by Curio Bioscience, Takara Bio), decodes the spatial information of the transcripts using DNA‐barcoded beads, with a resolution of 10 μm, which is near‐single‐cell level.^7^ The decreasing cost of next‐generation sequencing (NGS), alongside the advances in throughput, capture area and resolution of ST technologies, has led to its widespread adoption in cancer research.

MERFISH (multiplexed error‐robust fluorescence *in situ* hybridisation), is an imaging‐based spatial transcriptomics technology that can image hundreds of RNA molecules using a combinatorial labelling approach.^8^ Xenium *In Situ* from 10× Genomics is another imaging‐based platform that utilises padlock probes, which are hybridised to tissue sections.^9^ The sample then undergoes multiple rounds of imaging and transcript signal decoding in the Xenium Analyzer.^9^ The GeoMx Digital Spatial Profiler (DSP) from NanoString Technologies utilises RNA probes conjugated to ultraviolet (UV)–cleavable oligonucleotide linkers.^10^ Here, fluorescent labelling with morphology markers assists with the selection of region‐of‐interest (ROIs; at a size of 100 or more cells), which are illuminated with UV light, cleaving the linker from the probe.^10^ The released oligonucleotides are quantified using the NanoString nCounter system or NGS.^10^ The transcriptomic data are mapped back onto each ROI, reconstructing spatially resolved transcriptomic information.^10^ CosMx Spatial Molecular Imager (SMI), also from NanoString Technologies, performs multiple rounds of hybridisation using ISH probes, followed by imaging, during which high‐resolution z‐stacked images are acquired.^11^ The capacity to visualise each transcript at sub‐cellular resolution, with relatively straightforward experimental workflows, has facilitated the rapid adoption of imaging‐based technologies into cancer research.

### Integrative computational frameworks for analysing spatial transcriptomics data

#### Data pre‐processing

The upstream processing in sequencing‐based ST analysis involves generating a gene‐expression matrix with aligned spatial coordinates from the raw data. To obtain this matrix, key steps include tissue image processing, alignment of sequencing files and integrating them with spatial locations. Commercial platforms such as Visium and Space Ranger automate most of this alignment and integration process.

For imaging‐based data, pre‐processing consists of image registration, identification of transcript spots and cell segmentation. To generate spatially resolved single‐cell data, the image requires segmentation, grouping detected RNA transcripts into individual cells. Various software packages support the cell segmentation process, such as BIDCell,^12^ SSAM^13^ and BiomedParse.^14^ These tools enable the accurate detection of cell boundaries so that ST data are linked to single‐cell structures for more accurate biological insights. StarDist^15^ defines cell shapes using star‐convex polygons that accurately localise cell nuclei with radial distances from central points to edge. It uses CNNs to learn image features and predict star‐convex polygons for detected cells. CellPose^16^ models cells with spatial ‘flows’ from cell centres, dividing diverse cell shapes including spherical and elongated forms. Its flexible CNN adapts to various microscopy imaging applications and can work with pre‐trained models or be fine‐tuned for complex segmentation tasks.

Despite rapid progress, cell segmentation still faces notable limitations. Performance varies widely across tissue types, imaging modalities, training datasets and cell morphologies.^17^ Many tools are developed for a single tissue or image modality and generalise poorly to other data. Practical challenges including variable image quality, weak or ambiguous boundaries, touching or overlapping cells, and heterogeneous appearance remain difficult even for state‐of‐the‐art models.^18^ Fully supervised deep‐learning methods also require high‐quality, instance‐level annotations, which are expensive and demand expert effort.^17^ Finally, segmenting high‐resolution microscopy and WSI imposes heavy memory and compute requirements; scaling to these resolutions often forces trade‐offs between model size and accuracy.^18^

#### Downstream analysis

Downstream analysis for ST extracts meaningful information with data normalisation to adjust for sequencing depth variations and technical noise, batch effect correction addressing experimental variability and dimensionality reduction techniques to reduce high‐dimensional data.^19^ Clustering methods group cells/spots with similar expression patterns to identify distinct tissue areas, while identification of spatial domain defines coherent regions with typical molecular signatures. Cell type identification and deconvolution determine cell types and their contributions to multi‐cell per spot. Advanced analyses include spatial trajectory inference to reveal developmental or pathological progression patterns and cell–cell interaction analysis to explore neighbouring cells' communication based on spatial proximity and gene expression relationships.

#### Batch effect correction

Batch effect correction is a necessary procedure in ST analysis that helps to ensure that observed differences in gene expression truly arise from biological variations rather than technical differences across batches. There are numerous tools available for this effective correction. Among these, Harmony,^20^ LIGER^21^ and Seurat^22^ are the most efficient tools in terms of runtime and performance, making them particularly suitable for large datasets and complex analyses.^23^

#### Dimensionality reduction and clustering

In ST data analysis, dimensionality reduction is for extracting biologically meaningful patterns while maintaining spatial relationships in a lower‐dimensional space. The popular traditional methods such as Principal Component Analysis (PCA), t‐Distributed Stochastic Neighbour Embedding (t‐SNE) and Uniform Manifold Approximation and Projection (UMAP) are widely applied for this purpose. More recent software such as Scanpy,^24^ Squidpy^25^ and SpatialPCA^26^ have been developed and optimised for ST data. scVI^27^ is a probabilistic model for scRNA‐seq data analysis, which was built on a hierarchical Bayesian framework and a deep neural network. It encodes each cell's transcriptome into a low‐dimensional latent space, then decodes it with a zero‐inflated negative binomial model. scVI also addresses key problems such as library size and batch effects.

Following that, clustering methods are applied to divide tissue data into biologically relevant clusters. Conventional algorithms such as Louvain clustering, Leiden clustering, k‐means clustering and hierarchical clustering are frequently used.^28^ However, more advanced approaches have been developed in recent years. BayesSpace,^29^ for instance, applies Bayesian statistical models, SC‐MEB^30^ utilises Hidden Markov Random Fields, and SpaGCN^31^ uses Graph Convolutional Networks (GCNs) for spatial clustering.

A benchmark by Andrew et al. evaluated 15 clustering methods, revealing that SpaGCN,^31^ Seurat‐LV (Seurat with Louvain clustering method) and Seurat‐LVM (Louvain with multi‐level refinement) showed the most clustering accuracy, with Seurat^22^ demonstrating remarkable robustness. Interestingly, in case of mis‐specified parameters, SpaGCN,^31^ SpaGCN^+^ (SpaGCN method with input histology data), and Giotto‐H^32^ displayed top accuracy. Tools such as Seurat^22^ and stLearn^33^ were also highlighted for their comprehensive software support and ease of use across various ST analysis applications.^34^

#### Spatial cell‐type annotation

Cell type annotation for ST data is essential for understanding tissue structure and cellular diversity within spatial contexts. A common approach integrates scRNA‐seq data as a reference, using datasets from the corresponding or related tissue type.^35^ By aligning gene expression profiles from ST spots with single cells, researchers infer likely cell types in each spatial spot through two primary approaches: mapping and deconvolution. Cell‐type mapping is also commonly used for image‐based technologies, employing tools such as Seurat for single‐cell resolution data and CellTrek^36^ for lower resolution data. Both use ML methods including co‐embedding and random forest to project individual cells onto spatial coordinates.

The deconvolution approaches are mainly applied in sequencing‐based techniques, which produce spatial barcoding data without single‐cell resolution, and so estimating relative contributions of each cell type in spatial spots is required. Various tools have been developed for deconvolution including SPOTlight,^37^ Cell2location^38^ and Tangram.^39^ CARD^40^ uses non‐negative matrix factorisation and incorporates spatial correlations in cell‐type distributions, allowing for reference‐free deconvolution. UniCell Deconvolve Base (UCDBase)^41^ is a pre‐trained DL model for cell‐type deconvolution across various RNA‐seq formats, enabling context‐free deconvolution and can be used as a global cell type feature extractor for transfer learning.

#### Spatial Pattern Detection

Identifying spatially variable genes (SVGs) allows understanding of spatial organisation through three main methods: statistical‐modelling‐based (effective but computationally expensive), machine‐learning‐based (computationally efficient but faces challenges with high‐dimensional data) and spatial‐grid‐based (often binarise expression). Trendsceek^42^ employs permutation tests but increases processing time, while SpatialDE^43^ uses automatic expression histology for better efficiency but may result in type I errors. NicheCompass^44^ applies graph‐based DL by integrating signalling pathway knowledge and cellular communication, enabling the identification of cell niches and SVGs.

#### Cell–cell interaction

Various analytical approaches have been applied on ST to identify cellular communications within spatial contexts. Tools such as GCNG^45^ examine gene interactions between neighbouring cells, while SVCA^46^ measures the impact of cellular interaction on gene expression. NICHES,^47^ HoloNet^48^ and SpaCI^49^ measure ligand–receptor expression profiles for mapping communication pathways, with SpaCI^49^ shows its particularly performance on detecting intricate cell interactions and identifying upstream transcription factors in active signalling pathways.^50^

#### Gene–gene interaction

Gene–gene interaction analysis uncovers regulatory networks, identifies co‐regulated pathways and maps gene functions within specific tissue regions. Key computational tools include GCNG^45^ for graph‐based modelling of spatial relationships and gene interactions, scHOT^51^ for detecting complex gene correlations and multi‐gene interactions through higher order structural analysis, and MISTy^52^ for analysing gene interactions across multiple spatial contexts. MESSI^53^ predicts gene expression influenced by spatial signalling interactions, SpatialDE^43^ identifies spatially co‐expressed genes across regions, and Giotto^32^ builds co‐expression networks with spatial information.

#### Spatial trajectory

The key goal of studying cancer progression is to model how cells transition from a less malignant state to a more malignant one (e.g. from pre‐cancerous lesion to invasive cancer, or from primary tumor to metastasis). Trajectory inference algorithms were applied to single‐cell data to arrange cells along a *pseudotime* axis representing progression.

Waddington‐OT is an approach using optimal transport (OT) for studying developmental time courses to infer ancestor–descendant distribution, fate probabilities and interpolates held‐out time point and model the regulatory programs.^54^ TrajectoryNet introduces the continuous normalising flows that allow modelling the expected paths of points over time. Optimal transport provides a unified approach to inferring trajectories that is applicable to both stationary and nonstationary system.^55^ StationaryOT derives the steady‐state setting, adding growth (birth–death) terms to the Fokker–Planck framework, discussing identifiability and the need for assumptions on the drift field.^56^ TIGON is a dynamic, unbalanced OT algorithm based on a dimensionless formulation based on Wasserstein–Fisher–Rao (WFR) distance. Thanks to the unbalanced OT model, TIGON can reconstruct dynamic trajectories and population growth simultaneously as well as the underlying gene regulatory network from multiple snapshots.^57^

Spatial trajectory analysis maps cellular state progression over time through dynamic gene expression changes, with tools such as StLearn^33^ employing pseudo‐spatial trajectory analysis and SPATA^58^ leveraging Monocle3 for pseudo‐time inference, and methods such as Slingshot,^59^ PAGA^60^ are also widely used. The core innovation of Monocle3 lies in its generation of a PAGA (Partition‐based Graph Abstraction) graph to establish robust inter‐community connections, which guides the partitioning of cells into connected components that form the foundation for developmental trajectory modelling. Following principal graph construction, Monocle 3 performs pseudotime inference by projecting each cell along the graph and assigning developmental time points relative to user‐selected root nodes, which can be chosen based on developmental stage or expression features. For pseudotime estimation, PAGA uses an extended version of Diffusion Pseudotime (DPT) that handles disconnected graphs by assigning infinite distances between unconnected components and applying random‐walk‐based metrics within each component. In StLearn, the core of the method involves calculating the pseudo‐time–space distance (dPTS) between clusters, combining gene expression distance (dPT) and spatial distance (dS) using a weighting factor ω. Using these distances, a spatial‐PAGA graph is constructed; this is a topology‐preserving, directed graph that captures all possible transitions between sub‐cluster.

#### 3D data analysis

Three‐dimensional data are created by aligning serial tissue sections from single samples for in‐depth tissue architecture exploration. STUtility^61^ uses the Iterative Closest Point algorithm for slice alignment and 3D visualisation; PASTE^62^ employs Wasserstein Optimal Transport (FGW‐OT) for 3D alignment.

#### Spatial proteomic technologies to dissect the tumor‐immune landscape

Mapping the SP landscape of tumors reveals crucial functional‐level information about cellular states, neighbourhoods, ligand–receptor interactions and heterogeneity across samples. Technologies such as GeoMx DSP and CosMx SMI support both ST and SP, whereas technologies such as Co‐detection by indexing (CODEX), imaging mass cytometry (IMC) and Multiplexed Ion Beam Imaging (MIBIscope) are dedicated SP technologies.

CODEX is a multiplexed imaging‐based SP technology, commercialised as PhenoCycler by Akoya Biosciences.^63^ DNA‐conjugated antibodies bind to proteins on the tissue, to which complementary oligonucleotides with fluorophores are added.^63^ Fluorescently labelled antibodies are also used in technologies such as Lunaphore COMET, which is based on sequential immunofluorescence (seqIF), and MACSima imaging cyclic staining (MICS), which employs cyclic immunofluorescence.^64^, ^65^ Instead of a fluorescent readout, IMC utilises heavy metal‐tagged antibodies for spatial protein detection, followed by image acquisition using laser ablation integrated with mass cytometry.^66^ MIBIscope also uses metal‐tagged antibodies, but unlike IMC, it utilises an ion beam to release the metal‐tagged ions, which are measured using a time‐of‐flight mass analyser.^67^ GeoMx uses antibodies attached to UV‐cleavable linkers for SP, and CosMx uses antibodies attached to oligonucleotide tags. As SP develops, it offers a promising avenue for identifying complex TME interactions driving clinical outcomes.

#### Computational approaches for spatial proteomics workflows

Initial steps in the analysis of image‐based SP data involve image preprocessing and segmentation to identify individual cells. Methods such as Cellpose^16^ are general‐purpose algorithms used for cell segmentation in imaging‐based ST, which can also be applied for SP. Following segmentation, the cell's phenotype is determined, and per‐cell features, such as protein expression levels and spatial coordinates, are measured. The compiled single‐cell dataset reflects the spatially resolved architecture of the TME.^68^

Spatial omics data complexity necessitates user‐friendly analysis and visualisation tools. Sopa^69^ provides a technology‐invariant, memory‐efficient pipeline for image‐based spatial omics data, facilitating segmentation, transcript/channel aggregation, annotation, and geometric/spatial analysis with web reports and visualiser files. The Bioconductor^70^ bioinformatics ecosystem expands to support SP with peer‐reviewed analysis software. HIT‐MAP^71^ and ImShot^72^ provide open‐source bioinformatics tools for peptide and protein annotation in spatially resolved bottom‐up proteomics. Public SP datasets (described in the next section) inform computational approaches and enable reproducibility.

## Spatial multi‐omics to decipher cancer progression and microenvironmental evolution

### Publicly available ST and SP dataset for cancer research

Publicly available ST datasets provide significant contribution to cancer research by preserving spatial information while profiling gene expression. Large‐scale resources such as HTAN,^73^ HCA^74^ provide multi‐omics spatial data. Additionally, HEST‐1k,^75^ and STimage 1K4M^76^ introduce DL‐based analysis tools on spatial imaging and transcriptomics integration. They serve as invaluable resources for advancing spatially resolved cancer research and precision medicine.

The single‐cell and ST atlases provide crucial information about the cellular complexity of normal and cancerous breast tissue, varying in scale, sample sources and biological focus.^77^, ^78^, ^79^, ^80^ They analyse TME interactions, immune–epithelial dynamics, cancer stem‐like cell behaviour and therapy response prediction, incorporating diverse sequencing technologies, spatial mapping and computational tools such as SCSubtype^77^ and InteractPrint.^78^ Depending on the biological applications, some atlases prioritise immune–tumor interactions and epithelial heterogeneity; others focus on spatial tissue organisation or hormonal regulation. Collectively, they offer a multi‐dimensional understanding of cancer biology, tumor heterogeneity and microenvironmental interactions, supporting precision oncology and personalised treatment strategies.

Along with ST, advances in SP have also led to the generation of large proteomics datasets. Prominent databases such as Aquila^81^ host 107 datasets covering both SP and transcriptomics from 30 different diseases. Another significant resource is the SODB,^82^ which includes 132 datasets encompassing both SP and ST data. These databases also offer built‐in analytical tools for conducting spatial analyses, such as spatial colocalisation, spatially cell–cell communication. Furthermore, scProAtlas^83^ compiles SP data derived from eight distinct spatial protein imaging technologies. Integration of SP and scRNA‐seq datasets within scProAtlas^83^ utilises the MaxFuse^84^ algorithm, which identifies similar cell pairs across modalities by analysing both shared and distinct cellular features.

In parallel, the development of pretrained foundation models, such as CellFM^85^ scFoundation^86^ on ST data, KRONOS^87^ on SP data and scGPT^88^ for spatial omics data, has opened a new era for multi‐task biological application with the limited downstream training data.

### Spatial dynamics of cancer initiation

Spatially resolved transcriptomic and proteomic information from pre‐cancerous to malignant samples reveals the cellular landscape and interactions driving cancer initiation. Spatial mapping of oesophageal squamous cell cancer (ESCC) development identified a ‘CAF‐epi’ niche with activated cancer‐associated fibroblasts (CAFs) and epithelial cells at the periphery of high‐grade intraepithelial neoplasia and ESCC tumor.^89^ This niche induced an immunosuppressive and pro‐tumorigenic TME during ESCC progression from low‐grade intraepithelial neoplasia to malignancy.^89^ During CRC tumorigenesis, as the tumor develops, myeloid cells express SIRPα and malignant cells express its ligand CD47, whose interaction may promote immune escape.^90^ IMC identified pro‐inflammatory HLA‐DR^+^ CD204^+^ macrophages in normal colon, whereas HLA‐DR^−^ CD204^+^ macrophages were present in carcinoma, demonstrating macrophage reprogramming and immune escape mechanisms favoring CRC development.^90^ Integrating single‐cell and spatial multi‐omics in early and late‐onset prostate cancer (EOPC and LOPC), identified distinct microenvironments driving prostate cancer progression depending on the age of onset.^91^ In EOPC, APOE^+^ SPP1^+^ TAM infiltration into malignant cells with high androgen response meta‐program (AR‐MP) activation could induce an immunosuppressive microenvironment.^91^ In contrast, in LOPC tumors, PDGFRA^+^ iCAFs downregulate AR‐MP activation of malignant cells, and elevate EMT, resulting in tumor progression.^91^ In HCC, despite harbouring shared progenitor cells with similar CNVs in cirrhotic and tumor lesions, the tumor TME shifts towards an immunosuppressive state.^92^ This immunosuppressive milieu is supported by tumor cell–exhausted CD8^+^ T cell interactions via the CD86‐CTLA4 and HAVCR2‐LGALS9 axis, reiterating the role played by the tumor cells in modifying the TME to favor its development.^92^

### Trajectory analysis for cancer progression

Recent research has merged scRNA‐seq with ST to learn more about cancer progression in the context of tissue structure. Cai et al. integrated these approaches to study DCIS and IDC regions in breast tumors, revealing evolutionary progression patterns of tumor cells.^93^ By applying pseudotime analysis, they ordered cells from DCIS‐like to IDC‐like states, while spatial mapping demonstrated expression gradients at tumor boundaries, suggesting that spatial migration from ducts into invasive tissue corresponds to specific gene expression changes that are associated with aggressive behaviour.

Wang *et al*. used the Monocle2 software to investigate the expression profiles of gamma‐delta T‐cell trajectories changed over time and to find different cell states in triple negative breast cancer.^94^ These cells play an essential part of the TME and have been highlighted as potential biomarkers and therapeutic targets. This software was also applied for analysing the developmental trajectory of CD4^+^ T cell in lung adenocarcinoma.^95^ In oesophageal squamous cell carcinoma, Monocle2 was used to build a pseudotime map of CD8^+^ T‐cell state trajectory from naïve to exhausted state.^96^

In the TME, trajectory analysis has been applied to track immune cell differentiation and activation states. For example, the macrophage polarisation trajectory illustrates the transition from pro‐inflammatory to pro‐tumorigenic phenotypes, along with the spatial localisation revealing where each activation state resides, providing insights into immune cell dynamics and their spatial patterns during cancer progression.

### Mapping spatial evolution of the TME during progression and metastasis

**Figure 2:**
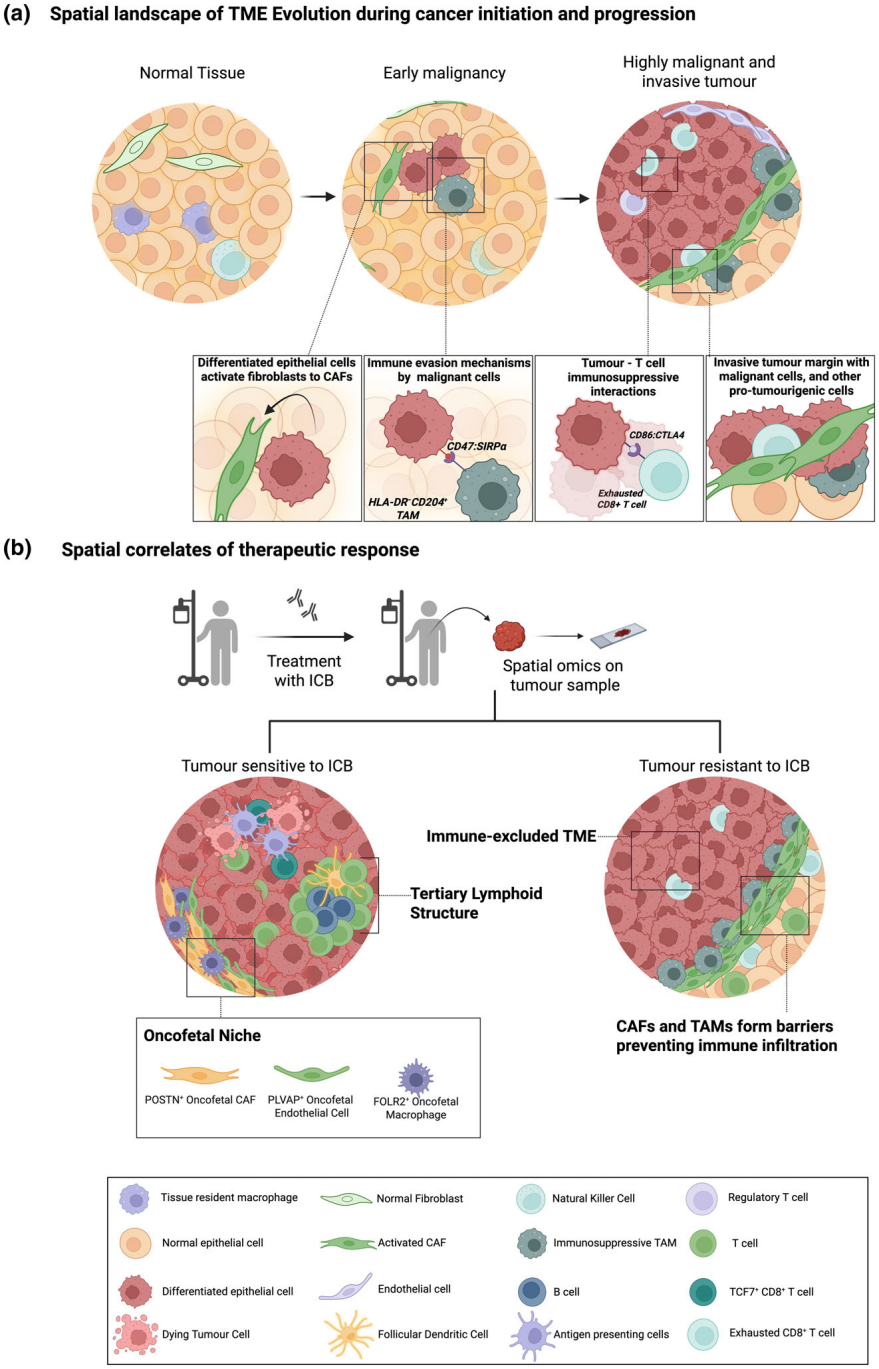
Spatial omics driven characterisation of the TME evolution. **(a)**, Performing spatial multiomics on tumor samples collected from different stages of cancer development (normal, pre‐malignant, and malignant tissues) enables the characterisation of the spatial landscape associated with tumor progression. This will reveal stage‐specific changes in the spatial architecture of the tumor and will aid in the identification of potential targets to prevent the lesion from becoming malignant. **(b)** Decoding the spatial biology behind therapeutic response by using spatially resolved technologies reveals the region‐specific ligand‐receptor interactions driving therapeutic response and the cellular architecture that correlates with response. A few examples of spatial correlates of therapeutic response are given. Tumors sensitive to ICB are often associated with the presence of TLS, increased TILs, with elevation of T cell populations such as TCF7^+^ CD8^+^ T cells and GZMB^+^ CD8^+^ T cells. An oncofetal niche consisting of POSTN^+^ CAFs, FOLR2^+^ TAMs and PLVAP^+^ endothelial cells has been identified to be associated with better response to combination immunotherapy in HCC. The spatial analysis of ICB resistant tumors from multiple cancer types has identified CAF subtypes forming physical barriers, which prevent the infiltration of immune cells into the tumor. ICB, immune checkpoint blockade, TLS, Tertiary Lymphoid Structure, TILs, Tumor‐infiltrating lymphocytes.

Spatial profiling of tumors across their developmental stages reveals stage‐specific cellular interactions driving progression (Figure 2a). By combining Visium and scRNA‐seq, a thyroid cancer study identified thyrocyte meta‐clusters with distinct immune and fibroblast composition dominating each stage of progression.^97^ SERPINE1‐PLAUR interaction between spatially localised fibroblasts and macrophages was elevated in highly aggressive cancer, suggesting their key role in progression.^97^ In CRC, CNV inference from ST data revealed spatially resolved clones and two pathways of progression—chromosome instability (CIN+) and hypermutated (HM).^98^ The epithelial cells of CIN+ tumors exhibited a high immune exclusion signature with low CD8^+^ T‐cell infiltration, showcasing a model where malignant cells induce an immune‐tolerant TME.^98^ A study investigating gastric adenocarcinoma progression identified two subgroups of intratumoral transcriptional heterogeneity, with distinct spatial localisation in the tumor.^99^ They also identified two evolutionary trajectories for gastric cancer, branched evolution and internal diaspora, each with distinct TME.^99^ Internal diaspora‐mediated evolution was linked to poor prognosis with a pro‐tumorigenic microenvironment consisting of VWF^+^ ACKR1^+^ endothelial cells and SPP1^+^ FN1^+^ tumor‐associated macrophages (TAMs).^99^ Another gastric cancer (GC) study identified inflammatory CAFs colocalising and communicating with cancer stem cells (CSCs) via the AREG/ERBB2 axis, upregulating CSC stemness and promoting progression.^100^ Expanding beyond 2D profiling, a recent pan‐cancer spatial multi‐omics study reconstructed the 3D tumor structure using spatial multi‐omics across serial sections, reiterating the spatial organisation of tumor evolution.^101^

The TME, consisting of non‐cancerous cells, extracellular matrix elements and various signalling factors, has emerged as a major driver in cancer development and treatment response. Its evolving role during multiple stages of tumor progression highlights it as a promising target for therapeutic intervention.^102^ During advanced BC, both malignant cells and the surrounding stroma undergo significant changes. Spatial studies have demonstrated that immune cells and fibroblasts exhibit dynamic phenotypic changes during tumor progression. A representative is macrophages, which play a dual role in tumor development. In early tumor stage, macrophages often exhibit a pro‐inflammatory (M1‐like) phenotype, which might constrain tumor growth. However, in more advanced tumors, they tend to transition to an M2‐like, immunosuppressive state that facilitates tumor progression and metastasis. ST enabled researchers to allocate distinct macrophage subsets to different tumor subregions and activation pathways. In a comprehensive multi‐modal analysis of BC progression, 37‐plex MIBI‐TOF SP was integrated with region‐specific Smart‐3SEQ transcriptomics and deep‐learning cell segmentation to link the evolving TME from normal tissue through DCIS to IBC.^103^ Early DCIS is characterised by myoepithelial hyperplasia and CD4^+^ T‐cell infiltration, whereas the progression to invasion features myoepithelial barrier loss, dense aligned fibrillar collagen deposition and proliferative CAF expansion. Longitudinal analysis of matched samples confirmed stromal remodelling as a key driver of progression.^103^

Fibroblasts also undergo significant alterations in the TME. For example, four distinct subpopulations of CAFs with varying spatial distribution were observed in an investigation on BC. Interestingly, iCAFs, with cytokine expression such as IL6, were more prevalent in aggressive BCs and were linked to an immunosuppressive microenvironment. This supports the idea that tumors actively recruit or redirect fibroblasts to evade immune responses. Besides, tumors with homologous recombination deficiency displayed a shift in CAF subtype dominance, with a higher prevalence of iCAFs over mCAFs, which correlated with altered immune cell function in these tumors. Spatial mapping revealed that iCAFs tended to cluster in tumor sites with low T‐cell infiltration and could be responsible for the formation of ‘cold’ immune‐excluded regions. These insights were derived through spatial gene expression analysis using nonnegative matrix factorisation and clustering techniques to deconvolute cell types, followed by immunostaining to confirm their localisation. A recent breast cancer (BC) study combined Visium and scRNA‐seq, identifying SPP1^+^ TAMs and TREM2^+^ TAMs colocalising with myofibroblastic FAP^+^ CAF subclusters, where the induction of TREM2^+^ TAMs generates an immunosuppressive milieu.^104^ An inflammatory FAP^+^ CAF subset with elevation of detoxification pathway genes was associated with FOLR2^+^ macrophages, forming an immunoprotective niche.^104^ In acral melanoma (AM), 22‐plex CODEX validated the spatial proximity between APOE^+^/CD163^+^ macrophages and tumor cells.^105^ It indicated that their interaction was mediated via the IGF1‐IGF1R axis through which TAMs induced EMT in the neighbouring tumor cells.^105^ Such studies underscore the power of ST in unravelling complex cellular interactions within tumors.

ST in BC enables the measurement of tumor‐associated immune cells, stromal cells and blood vessels surrounding tumors, opening potential targets for precision therapies.^106^ Rania Bassiouni et al. report a key finding concerning the association between immune cells and the TME was that stratification of samples by race revealed race‐associated differences in regions of immune‐rich infiltrate.^107^ This indicates that the composition of the TME, specifically the proportion of immune cells, can vary based on race, and the study identified these proportional differences in tumor cell composition by race through innovative computational approaches.

Elucidating the differences between the tumor core and periphery can uncover region‐specific mechanisms, otherwise obscured in bulk or single‐cell analysis. Using Visium on primary liver cancer (PLC), a study identified cell clusters with unique gene expression patterns, spatially localised in different tumor regions.^5^ In particular, a PROM1^+^ cancer stem cell niche increased in abundance from the tumor margin towards the core, with its highest abundance in the portal vein thrombus region.^5^ PROM1^+^ CSC niches had elevated gene expression for pathways such as hypoxia, epithelial‐mesenchymal transitions (EMT) and tumor necrosis factor‐a (TNF‐α), indicating their potential to modify the TME.^5^ In another PLC study, Stereo‐seq identified invasive tumor cells secreting serum amyloid A1 and A2 at the tumor border and recruiting FPR1^+^ macrophages to induce an immunosuppressive niche.^108^ In intrahepatic cholangiocarcinoma (ICC), CD8^+^ MAIT cells at the invasive border, recruit FOLR2^+^ macrophages, which develop into SPP1^+^ macrophages.^109^ These pro‐tumorigenic SPP1^+^ macrophages colocalise with endothelial cells and POSTN^+^ FAP^+^ fibroblasts, forming a triad, where the macrophages adapt a pro‐angiogenic role.^109^ Similarly, fibroblasts and myeloid cells were abundant at the periphery of aggressive thyroid cancer, and SPP1^+^ immunosuppressive macrophages also accumulated at the periphery of cervical squamous cell carcinoma.^97^, ^110^ When compared to late‐onset colon cancer, the invasive tumor margin in early‐onset colon cancer (EOCC) was enriched in FAP^+^ CAFs with elevated WNT signalling pathway and distinct epithelial subsets.^111^ Their crosstalk mediated by the FGF20‐FGFR2 axis could play a role in tumor progression at the invasive margin, thereby highlighting a potentially targetable stromal‐epithelial interaction in EOCC.^111^

In HCC, GeoMx whole transcriptome DSP, combined with single‐cell and bulk technologies, showed a selective metastatic advantage for certain subclones.^112^ These subclones had wild‐type Wnt, harboured terminally exhausted CD8^+^ T cells, inflammatory CAFs and invasive margin tumor cells, offering them a higher metastatic capacity.^112^ Distinct spatial ecotypes were identified in the liver and lymph node metastasis of pancreatic ductal adenocarcinoma, with the primary tumor ecotypes being highly desmoplastic, enriched with myCAF, Tregs and immunosuppressive TAMs.^113^ Conversely, the metastatic tissue ecotypes had less stromal content, higher abundance of malignant cells and upregulated glycolysis and proliferative pathways.^113^ Another study identified a specific population of Notch4‐driven macrophages that were enriched in metastasis‐prone tumors. These macrophages, spatially localised in invasive niches and lymph node metastases, had an elevation of genes associated with immune suppression.^106^ Spatial analysis of BC lymph node metastases revealed macrophages highly enriched for NF‐κB signalling and cytokine production pathways, suggesting their role in creating a supportive environment for tumor cell colonisation. The spatial differentiation trajectory of macrophages showed their transition from a chemokine‐producing to an antigen‐presenting state within tumor‐infiltrated lymph nodes, indicating that TAMs gradually acquire new functions as they interact with tumor cells and lymphocytes in metastatic sites, further influencing disease progression. Another study combined slide‐seqV2 and snRNA‐seq in primary tumors and brain metastasis from NSCLC.^114^ In brain metastasis, genes associated with TGF‐β signalling, EMT and MYC target expression were upregulated in myeloid‐rich regions, whereas in the primary tumor, high myeloid density was associated with interferon responses and antigen presentation.

### Spatial evolution under therapy and the clinical translation of spatial omics

Applying spatial omics technologies to samples from different treatment stages uncovers the influence of the TME evolution on treatment outcomes and has the potential to identify spatial response biomarkers. Moreover, this enables the identification of spatial correlates of immunotherapy response (Figure 2b). Various biomarkers have been investigated for immunotherapy response, with tertiary lymphoid structures (TLS) emerging as a significant predictor of response.^115^ Novel spatial technologies offer ways to improve the characterisation of these spatially localised immune aggregates, enabling better patient stratification before immunotherapy. In TNBC, a 30‐gene TLS signature associated with immunotherapy response was identified by combining histopathological information with spatial transcriptomics.^116^ Similarly, in head and neck squamous cell carcinoma, CODEX identified a TLS neighbourhood with a high abundance of CD38^high^ CD31^high^ plasma cells, associated with better RFS after surgery.^117^ The presence of FAP^+^ ASMA^+^ CAFs and MYH11^+^ ASMA^+^ CAFs is associated with poor immunotherapy response in NSCLC despite the presence of TLS.^118^ Inflammatory FAP^+^ ASMA^+^ CAFs promoted the exhaustion of CD8^+^ T cells, whereas MYH11^+^ ASMA^+^ CAFs induced immunosuppression by increasing Treg infiltration.^118^ In a recent BC study, Xenium revealed a subset of fibroblasts expressing CCL21 and APOD adjacent to TLS^119^. This study also revealed interactions along the CXCL12/CXCR4 axis in regions with TLS and CCL21^+^/APOD^+^ fibroblasts, demonstrating spatially localised chemokine interactions through which CAFs perform lymphocyte recruitment.^119^

Combining Stereo‐seq with scRNA‐seq, the expansion of SLIT2^+^ CAF and PI16^+^ CAF subsets was identified following neoadjuvant chemotherapy in rectal cancer patients.^120^ These CAF subsets colocalised with CD8^+^ Trm, CD8^+^ Tem, and conventional DC2s in complete responders.^120^ Alternatively, in non‐responders, FAP^+^ CAFs and BMP4^+^ CAFs colocalised with SPP1^+^ macrophages.^120^ In a head and neck carcinoma study (HNC), CosMx SMI and GeoMx DSP were integrated to map the effects of Subasumstat as neoadjuvant treatment.^121^ SMI revealed drug‐induced changes in the TME, including the activation of IFN signalling, elevated expression of CXCL10 in fibroblasts and macrophages, and a shift in macrophage polarisation state from M2 to pro‐inflammatory M1 subtype.^121^ Macrophages expressing CXCL10 were observed to be spatially correlated with cytotoxic T cells expressing Granulysin (GNLY).^121^ These observations were present in regions with target engagement by the drug, indicating drug‐induced changes in the TME.^121^ GeoMx DSP was also employed to characterise the association between the spatial proteomic landscape and response to anti‐PD‐1 treatment in CRC patients.^122^ This study utilised a 71‐plex protein panel and identified that patients with shorter progression‐free survival (PFS) had elevated fibronectin and smooth muscle actin (SMA) in the epithelial compartment of the tumor.^122^ In the CD45^+^ immune compartment, CD27, ICOS, Ki‐67 and GZMA elevation were associated with longer PFS.^122^ Similarly, IMC on ESCC samples identified a subpopulation of CD39^+^ PD‐1^+^ CD8^+^ T cells (precursor exhausted T cells), positively correlating with ICB response.^123^

Another study identified ‘oncofetal niches’ in HCC, consisting of cell types shared between tumor and fetal tissue, but absent in healthy adult liver.^124^ In 2020, Sharma et al. discovered an oncofetal ecosystem in the TME of HCC, consisting of PLVAP^+^ endothelial cells and FOLR2^+^ TAMs.^125^ In 2024, oncofetal CAFs expressing periostin (POSTN^+^ CAFs) were discovered as the third component of the oncofetal ecosystem in HCC.^124^ A higher oncofetal score, reflecting a greater abundance of oncofetal cells, was linked to a risk of early relapse after surgical resection.^124^ Moreover, tumors with a higher oncofetal score responded better to combination therapy with atezolizumab (anti‐PD‐L1) and bevacizumab (anti‐VEGF), offering an opportunity for patient stratification, before combined immunotherapy^124^ (Figure 2b). An upcoming clinical trial (DEFINERx050—HREC reference number: 2024/ETH02271) will investigate this systematically, where patients with HCC tumors harbouring a high oncofetal signature will be randomised to receive either surgical resection directly or receive neoadjuvant immunotherapy prior to surgery. This trial could demonstrate the advantage of oncofetal signature as a biomarker to identify HCC patients at risk of relapse after surgery, who may benefit from neoadjuvant immunotherapy.

In pre‐treatment samples from lung cancer patients, combined spatial transcriptomics and proteomics identified the presence of a ‘stem immunity hub’ associated with anti‐PD‐1 response.^126^ This cellular hub was characterised by TCF7^+^ CD8^+^ T cells, elevated CXCL10/CXCL11 expression, CCL19^+^ fibroblasts, mregDCs and CXCL10^+^ macrophages.^126^ In HCC patients undergoing neoadjuvant immunotherapy, spatially colocalised cellular triads consisting of mregDCs expressing LAMP3, PD‐1^high^ CD8^+^ T cells, and CXCL13^+^ T helper cells were enriched in responder patients.^127^ These cellular niches could be responsible for the expansion of progenitor CD8^+^ T cells into antitumor CD8^+^ T effector cells, thereby enhancing immunotherapy response.^127^ Cellular triads formed by CD4^+^ T cell, CD8^+^ T cell and antigen‐presenting cells were also higher in immunotherapy responders in pleural mesothelioma patients.^128^

Spatial technologies have enabled the identification of desmoplastic barriers by CAFs, which hinder T‐cell infiltration.^129^ Combined single‐cell and spatial transcriptomics studies in HCC revealed an increased positive correlation between POSTN^+^ CAF and SPP1^+^ macrophages in non‐responder patients, contributing to an immune‐excluded TME, dampening immunotherapy response.^130^ POSTN^+^ CAFs and SPP1^+^ TAMs forming physical barriers preventing immune infiltration have also been observed in ICC and NSCLC.^109^, ^131^ In intrahepatic cholangiocarcinoma (iCCA), CODEX with a 38‐marker panel was used for spatial immunophenotyping.^132^ In FGFR2 altered iCCA, high abundance of CD11b^+^/CD15^+^ polymorphonuclear myeloid‐derived suppressor cells was identified along with reduced abundance of CD8^+^ T cells, demonstrating the presence of suppressive myeloid cells in the TME along with low T‐cell infiltration.^132^ The TME landscape of IDH1 altered iCCA, showed strong interactions of tumor cells with fibroblasts and CD68^+^/CD163^−^ macrophages, potentially forming barriers for T‐cell infiltration and therapeutic drugs.^132^ In oral squamous cell carcinoma, STMN1^+^ capillary endothelial cells and MYF5^+^ muscle satellite cells created an immunosuppressive niche by colocalising and interacting with tumor cells through TGF‐B and NOTCH signalling in non‐responders to immunotherapy.^133^ However, in responders, pro‐immunogenic SELP^+^ HEV‐like endothelial cells and APOD^+^ myCAFs colocalised with MHC‐II^+^ T/NK cells, highlighting their role in enhancing immune infiltration and improving therapy response.^133^

Given the TME's role in shaping treatment response, its characterisation offers a promising avenue for discovering predictive response biomarkers. A melanoma study used cytometry time‐of‐flight (CyTOF) IMC and identified that higher Ki67 expression on CD45RO^+^ antigen‐experienced T cells in pre‐treatment samples correlated with better response to ICI.^134^ In responders, antigen‐experienced T cells were physically closer to melanoma cells, highlighting the importance of the spatial immune architecture in immune checkpoint inhibitor (ICI) response.^134^ Similarly, in a TNBC study, GeoMx DSP identified the correlation between the immune landscape in early‐stage treatment‐naïve samples, identifying that high intra‐epithelial HLA‐DR and CD40 expression is associated with longer recurrence‐free survival (RFS) to systemic treatment.^135^ In HER2‐positive breast cancer, spatial proteomics revealed biomarkers of pathological complete response (pCR) towards neoadjuvant HER2‐targeted therapy.^136^ This study, conducted on biopsies collected from the TRIO‐US B07 trial, demonstrated proteomic changes in Ki‐67, HER2, and immune markers shortly after treatment, which served as predictors of pCR.^136^ In treatment‐naïve TNBC samples, the proportion of proliferating MHC‐II^+^ cancer cells and CD8^+^ TCF7^+^ T cells was a predictor of response to neoadjuvant immunotherapy.^137^ Moreover, elevated CD8^+^ GZMB^+^ T cell densities in on‐treatment samples indicated potential response, while epithelial cell interaction with CD79a^+^ plasma cells indicated resistance to ICB.^137^ In HCC tumor biopsies, CODEX revealed four immune classes, and the immune class associated with the best survival showed increased interactions between CD4^+^ T cells, CD8^+^ T cells, and tumor cells.^138^ Increased spatial proximity between CD68^+^ TAMs and T cells, and elevated CD68^+^ TAM–tumor cell interactions were associated with poor response.^138^ The ability to link clinical outcomes with the spatial proteomic landscape in biopsies offers a translatable tool for predicting response and guiding treatment decisions.^138^

## Leveraging spatial omics and AI to decode tumor microenvironment dynamics across cancers

**Figure 3:**
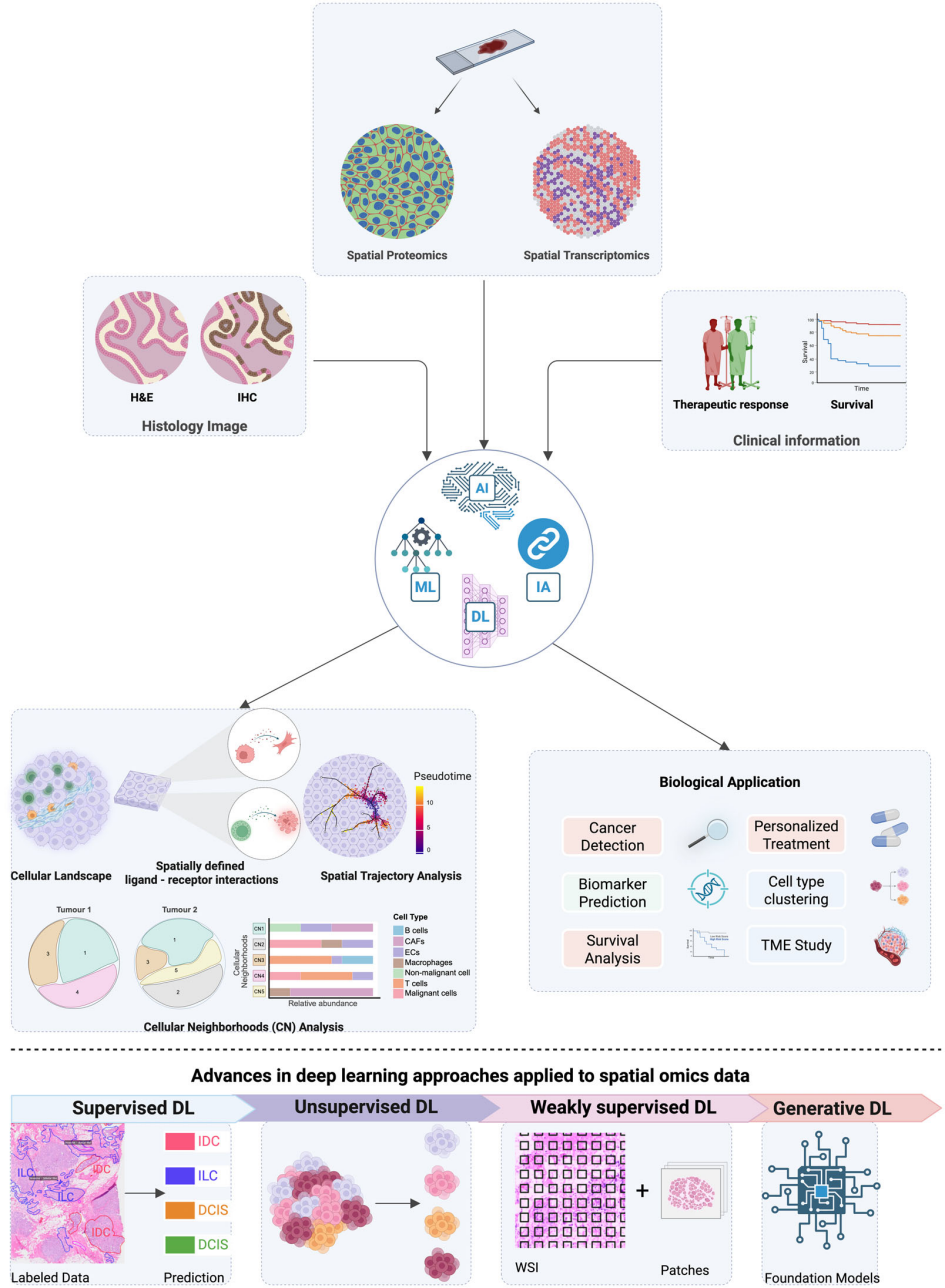
AI‐Enabled Multi‐omics for Cancer Translational and Clinical Research. AI‐driven integration of different modalities, including (i) histology images (H&E/IHC whole‐slide images), (ii) spatial omics (spatial transcriptomics, spatial proteomics), and (iii) clinical metadata (treatment response and survival data) are jointly modelled with integrative analysis (IA), artificial intelligence (AI), machine learning (ML), and deep learning (DL). Central models learn representations across modalities to support downstream spatial analyses, including reconstruction of cellular landscapes, inference of spatially defined ligand–receptor interactions, cellular neighbourhood (CN) profiling, and trajectory/pseudotime analysis. Insights generalise to biological and clinical applications such as cancer detection, biomarker prediction, survival analysis, cell‐type clustering, tumor‐microenvironment (TME) studies, and personalised treatment selection. The bottom timeline shows the evolution of deep learning approaches.

As spatial data is highly complex, advances in spatial analyses have enabled the discovery of spatial patterns from this multimodal data, with limited sample size. In this session we discuss how AI, ML and IA are used (Figure 3).

Atezolizumab–bevacizumab response signature (ABRS) is related to progression‐free survival after treatment initiation of the hepatocellular carcinoma patients. ABRS‐P has been developed to predict the ABRS expression from the histopathology image. The model is built on CLAM‐style attention multiple‐instance learning (MIL): Patches are encoded with CTransPath, attention weights are learned for each patch, and an attention‐weighted aggregation of patch‐level regression outputs yields the whole‐slide ABRS‐P.^139^ Julien et al. also utilised attention‐based MIL framework to aggregate the bag of feature vector by ResNet50 via a softmax‐weighted sum to produce a single slide‐level representation.^140^ A final fully connected layer mapped this representation to class probabilities. This model demonstrated the ability to classify hepatocellular carcinoma (HCC) and intrahepatic cholangiocarcinoma (ICCA).

The stepwise malignant progression from normal tissue through precancerous leukoplakia to fully developed cancer uncovers early epithelial and immune changes before overt malignancy.^141^ Further analysis of the TME evolution by reconstructing cellular progression trajectories reveals intensifying immune suppression over time, driven by distinct immunoregulatory cell populations, including LAMP3^+^ dendritic cells and M2 macrophages, which contribute to immune evasion.^142^ Both studies underscore the power of single‐cell technologies in capturing the dynamic interplay between cancer cells and the TME, offering valuable insights into early detection and therapeutic targeting.

The convergence of ML techniques with ST is reshaping current insights into tumor progression, microenvironmental interactions, and the molecular characteristics of pancreatic cancer. A recent study applied autoencoder for spectral denoising and CNN for tissue classification to high‐resolution molecular imaging datasets, enabling the detection and semi‐quantitative analysis of spatial epigenetic patterns across pancreatic cancer subtypes.^143^ Their findings uncovered distinct chromatin modifications and spatial diversity linked to tumor aggressiveness, suggesting new avenues for identifying potential. Another study used ST to identify the TME evolution from primary pancreatic ductal adenocarcinomas to metastatic site.^113^ This revealed notable disparities in the spatial distribution of immune and fibroblast populations, particularly an enhanced immune exclusion in hepatic metastases and localised expression of genes promoting metastasis.

Bai et al. demonstrated an innovative approach by combining multi‐omics analysis with ML to elucidate the influence of programmed cell death (PCD) mechanisms in the progression of gastric cancer. Through transcriptomic profiling of various PCD pathways, including apoptosis and ferroptosis, they categorised gastric tumors into three molecular subtypes. Their ML framework integrates 10 algorithms, namely CoxBoost, stepwise Cox, Lasso, Ridge, elastic net, survival support vector machines, generalised boosted regression models, supervised principal components, partial least squares Cox, and random survival forest, enabling patient stratification based on risk, revealing that individuals exhibiting increased immune cell infiltration and elevated expression of pivotal PCD‐associated genes had notably improved survival outcomes. This underscores the prognostic relevance of PCD gene signatures and effectively links molecular alterations with immune microenvironment characteristics, offering valuable insights for the advancement of personalised treatment strategies in gastric cancer.^144^

## Challenges and future directions

Despite significant advancements, several challenges remain, particularly in the computational analysis of spatial omics data. Compared with bulk RNA‐seq and scRNA‐seq, the number of computational tools for spatial omics remains limited, especially for spatial proteomics.^145^ Moreover, spatial omics assays are generally more expensive, requiring specialised instrumentation and advanced computational infrastructure. Data quality is highly sensitive to tissue handling, sectioning and preservation protocols, and batch effects can impact the robustness and reproductivity of spatial analyses.^146^ While single‐cell technologies lack spatial context, they typically provide broader or whole genome coverage, whereas spatial transcriptomics technologies involve trade‐offs between spatial resolution and molecular coverage across platforms. Integrating datasets from different spatial omics technologies is hindered by incomplete data, differences in panel design, varying resolution, signal strength, and differences in analytical methodologies. Moreover, processing spatially resolved data requires substantial computational power and storage, particularly for AI‐driven applications. Enhancing the use and interpretability of spatial omics‐based AI models is of key importance in translating the results to clinical use. Emerging computational tools and pipelines should focus on addressing the current challenges and should improve the capacity to integrate results from different spatial omics platforms.

Although current spatial omics research primarily focusses on discovery, the insights should be further explored in pre‐clinical studies and clinical trials.^147^ ST and SP can enable the identification of spatial biomarkers, informing the timely selection of appropriate therapy. These tools can stratify tumors based on spatially resolved gene expression, cellular landscapes or the presence of immune hubs, all of which are associated with treatment outcome. Further research and development are needed to create standardised experimental workflows, analysis pipelines and cost‐effective approaches, which are essential before translating these technologies into a routine pathological setting. Continued collaboration between algorithm developers and translational researchers bridges the gap between complex spatial omics data and impactful clinical practice. Nevertheless, this field is progressing rapidly and is well‐poised to be integrated into the next generation of pathological tests for immuno‐oncology.

## Author Contributions

**Hao Nguyen:** Conceptualisation, writing – original draft, writing – review and editing. **Merrin Mary Eapen:** Conceptualisation, writing – original draft, writing – review and editing. **Quan Nguyen:** Conceptualisation, supervision, writing – review and editing. **Ankur Sharma:** Conceptualisation, supervision, writing – review and editing.

## Conflict of Interest

The authors declare no conflicts of interest.

## Data Availability

Data sharing not applicable to this article as no datasets were generated or analysed during the current study.
